# Enhancing Capillary Pressure of Porous Aluminum Wicks by Controlling Bi-Porous Structure Using Different-Sized NaCl Space Holders

**DOI:** 10.3390/ma17194729

**Published:** 2024-09-26

**Authors:** Hongfei Shen, Asuka Suzuki, Naoki Takata, Makoto Kobashi

**Affiliations:** Department of Materials Process Engineering, Graduate School of Engineering, Nagoya University, Furo-cho, Chikusa-ku, Nagoya 464-8603, Japan; shen.hongfei.f4@s.mail.nagoya-u.ac.jp (H.S.); takata.naoki@material.nagoya-u.ac.jp (N.T.); kobashi.makoto@material.nagoya-u.ac.jp (M.K.)

**Keywords:** capillary pressure, permeability, space holder, bi-porous structure, aluminum foam

## Abstract

Capillary pressure and permeability of porous media are important for heat transfer devices, including loop heat pipes. In general, smaller pore sizes enhance capillary pressure but decrease permeability. Introducing a bi-porous structure is promising for solving this trade-off relation. In this study, the bi-porous aluminum was fabricated by the space holder method using two different-sized NaCl particles (approximately 400 and 40 μm). The capillary pressure and permeability of the bi-porous Al were evaluated and compared with those of mono-porous Al fabricated by the space holder method. Increasing the porosity of the mono-porous Al improved the permeability but reduced the capillary pressure because of better-connected pores and increased effective pore size. The fraction of large and small pores in the bi-porous Al was successfully controlled under a constant porosity of 70%. The capillary pressure of the bi-porous Al with 40% large and 30% small pores was higher than the mono-porous Al with 70% porosity without sacrificing the permeability. However, the bi-porous Al with other fractions of large and small pores did not exhibit properties superior to the mono-porous Al. Thus, accurately controlling the fractions of large and small pores is required to enhance the capillary performance by introducing the bi-porous structure.

## 1. Introduction

Open-cell porous metals are a type of metal foam characterized by interconnected pores that allow for fluid flow and exhibit unique properties such as high specific surface area per volume, fluid permeability, and lightweight structure. These characteristics enhance their performance in various applications such as catalysis, filtration, and thermal management [1,2,3]. Their versatility extends to fields like medical implants, energy storage, and environmental engineering [4,5]. The ability to customize their porosity makes open porous metals adaptable for specific needs, driving innovation and utility across diverse industries.

Open-cell porous metals are promising wick materials in the loop heat pipes [6,7,8]. In the LHP, capillary pressure and permeability play crucial roles in the efficient transport of heat. LHPs rely on the capillary action of a porous wick structure to drive the working fluid from the evaporator to the condenser without requiring mechanical pumps. Capillary pressure, generated by the surface tension between the liquid and the solid wick, enables the fluid to overcome gravitational and frictional resistance, ensuring continuous heat transfer. Permeability, a property of the wick material, influences the ease with which the fluid can move through the porous structure. High permeability enhances fluid flow, reducing pressure losses and improving overall thermal performance. Therefore, optimizing both capillary pressure and permeability is essential for enhancing the efficiency and reliability of LHPs in thermal management systems, particularly in space and electronics cooling applications [9,10]. Numerous studies have focused on finding porous structures with high capillary pressure and permeability [11,12]. The pore size is an important factor for the capillary pressure and permeability [13,14]. The following formula expresses the capillary pressure (Δ*P*_cap_).

(1) $$\Delta P_{\mathrm{cap}}=\frac{2\sigma_{L}\cos\theta }{r}$$

Here, *σ*_L_ is the surface tension of the liquid (the force that causes the surface of a liquid to contract), *q* is the contact angle (the angle where a liquid meets a solid surface, indicating how well the liquid wets or spreads on that surface), and *r* is the flow channel radius. *r* correlates with the pore size in the porous wicks, and the capillary pressure can be enhanced by reducing the pore size. However, the reduced pore size resulted in a large flow resistance and low permeability. The permeability (*K*) is expressed in the following formula.

(2) $$K=\frac{\varepsilon D_{e}^{2}}{32{\left(L_{e}/L\right)}^{2}}$$

Here, the *e* is the porosity, *L*_e_/*L* is the tortuosity (a measure of how convoluted or winding a path is within a material, affecting the ease with which fluids can flow through it), and *D*_e_ is the flow channel diameter. *D*_e_ also correlates with the pore size in the porous wicks, and the permeability is decreased by reducing the pore size [15]. Thus, the permeability and capillary pressure have a trade-off relation via the pore size. A solution to the trade-off relation between the permeability and capillary pressure is to introduce bi-porous structures with bi-modal pore size distribution. Many experimental and computational studies have demonstrated that the bi-porous structure exhibited superior capillary performance compared to the mono-porous structure [16,17,18]. Liquid preferentially infiltrates into smaller pores and wets the cell walls of larger pores, improving the capillary pressure.

Many researchers have attempted to prepare bi-porous materials via combining the space holder method, which has a high controllability of porous structure, and another process. Cao [19,20] fabricated bi-porous Ti_3_AlC_2_ wicks via sintering Ti/Al/TiC powder mixtures with a sodium chloride (NaCl) space holder, which is dissolvable in water. The bi-porous structure consisted of fine gap pores (<2 μm) and large pores formed by space holders (6–12 μm). Zhu [21] produced hierarchical porous Cu containing both micro- (200–450 μm) and nanopores (200–600 nm) through combining a K_2_CO_3_ space holder and chemical dealloying [22]. Shu [23,24] manufactured porous NiAl intermetallic compounds with a bi-porous structure via combining the combustion synthesis reaction [25] and the NaCl space holder method [26]. Small pores with several micrometer sizes were formed by the Ni-Al combustion synthesis reaction. However, these processes stochastically form small pores and cannot intentionally control their fraction and sizes, resulting in a poor understanding of suitable bi-porous structures (e.g., fractions of large and small pores). One promising approach for intentionally controlling the bi-porous structure is the space holder method using space holder particles of different sizes. It is expected that the fraction and size of large and small pores can be controlled independently through tailoring the fraction and size of two space holder particles. Evaluating the capillary performance of bi-porous materials controlled by the two different-sized space holder methods leads to a better understanding of suitable bi-porous structures for the porous wicks.

In our previous study [27], sodium chloride (NaCl) powders of around 400 μm were applied as the space holder to prepare porous aluminum (Al) with a different porosity. Increasing porosity led to higher fluid permeability and lower capillary pressure even though the pore size barely changed. This was because connective parts between adjacent pores were widened by increasing porosity. The connective parts had a small flow channel size, and its widening increased the permeability and decreased the capillary pressure. Thus, the porous Al fabricated via the space holder method also exhibited the trade-off relation between the capillary pressure and fluid permeability, which was indirectly affected by the pore fractions. In this present study, bi-porous Al was fabricated using NaCl space holder particles of two different sizes. The effect of the volume fractions of small and large NaCl particles on the porous structure and the capillary performance was systematically investigated under a constant total volume fraction of NaCl, e.g., constant total porosity. These results were used to discuss suitable bi-porous structures for enhancing capillary performance.

## 2. Materials and Methods

### 2.1. Space Holder Method

**Figure 1:**
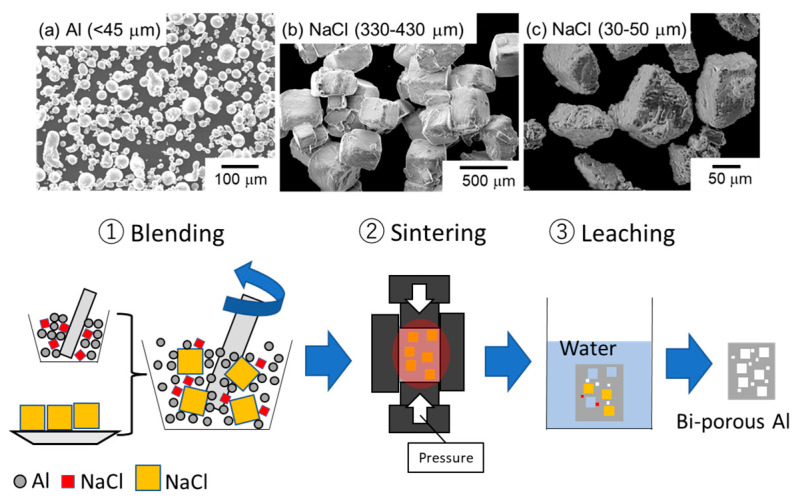
SEM images of raw materials and fabrication process of bi-porous Al using space holder method.

Figure 1 shows scanning electron microscope (SEM) images of the raw materials and the fabrication process of bi-porous Al using the space holder method. Powders of aluminum (≤45 μm, 99.99%) and NaCl particles with different sizes (30–50 μm and 330–430 μm) were used. Different combinations of Al powder and NaCl particles considered in this study are summarized in Table 1. In the mono-porous group, only small NaCl particles were blended with aluminum so that the NaCl volume fractions were set at 50%, 60%, and 70%. Volume fractions greater than 80% cannot maintain the skeleton after dissolving in water, while lower volume fractions cannot ensure the complete dissolution of NaCl. In the bi-porous group, the total volume fraction of NaCl was kept constant at 70% for the same reason, while the volume fraction of small NaCl particles was increased from 10% to 40%. This also allows us to compare them with the counter mono-porous sample under the same total porosity. The samples were labeled using L (large), S (small), and the volume fractions. For example, the porous sample fabricated using the large NaCl of 60% and the small NaCl of 10% was denoted as L60S10. Al and NaCl were manually blended in a bowl with a ceramic bar for 10 min, twice and placed into a cylindrical graphite mold (inside diameter: 10 mm, outside diameter: 20 mm, height: 50 mm), followed by spark plasma sintering (CSP-KIT-02121, PLASMAN, Hiroshima, Japan) under vacuum conditions (~10 Pa). The temperature was increased at a heating rate of approximately 0.5–0.67 °C/s and kept at 600 °C for 600 s under a constant applied pressure of 20 MPa. The sintered samples were placed in running water for over 86.4 ks (24 h) to completely remove the NaCl particles. The samples were put into a hot dryer and heated at 150 °C for 10.8 ks (3 h).

The mass, diameter, and height of each sample were measured to quantify the total porosity (ε) [28].

(3) $$\varepsilon =1-\frac{\rho_{\mathrm{sample}}}{\rho_{\mathrm{Al}}}=1-\frac{4m_{\mathrm{leached}}}{\pi d^{2}h\rho_{\mathrm{Al}}}$$

Here, *ρ*_sample_ is the bulk density of the sample after NaCl removal, *m*_leached_ is the mass of the samples after NaCl removal, *ρ*_Al_ is the true density of Al (2.7 g cm^−3^), and *d* and *h* are the diameter and height of the cylindrical sample (*d* = 10 mm, *h* = 19~23 mm). The quantified porosity is summarized in Table 1 and is well consistent with the designed total porosity of each sample. The porous samples were immersed in epoxy resin and polished with SiC abrasive papers and diamond slurries of 3 μm and 1 μm in particle sizes. The porous structures were observed using an SEM (JSM-IT500, JEOL, Tokyo, Japan). Image analysis using software (Image J 1.53t, National Institutes of Health, Bethesda, MD, USA) was conducted to estimate porosity and pore size.

### 2.2. Capillary Rise Experiment

**Figure 2:**
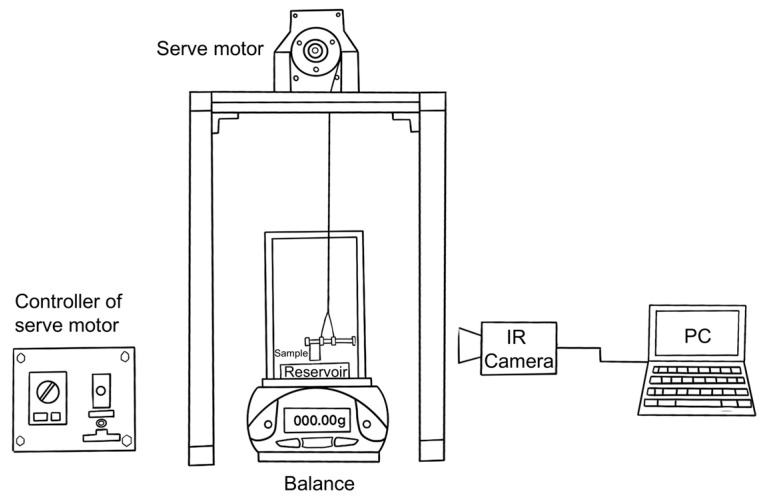
Schematic illustration of setup for measuring the capillary performance of porous samples in this study [27].

Figure 2 shows a schematic of the experiment setup. The cylindrical sample (diameter: 10 mm, height: 19–23 mm) was contacted vertically to the water reservoir, and then the water was spontaneously infiltrated upward into the porous sample. Infrared (IR) thermal imaging was utilized to capture the water height during the capillary rise processes. The infrared camera (TVS-500EX, Nippon Avionics, Yokohama, Japan) with a temperature resolution better than 0.05 K and an accuracy of 2% for temperatures below 300 °C was used. The detailed measurement method is given elsewhere [27]. Before the experiments, all the test samples were dried for 1.8 ks (0.5 h). The tests were conducted at an ambient temperature of 22 ± 1 °C. To reduce test errors, each sample was tested three times, and the average value was taken as the final result.

According to Darcy’s law, liquid is driven by capillary pressure and countered by viscous resistance and gravity in the capillary wicking process [29].

(4) $$\Delta P_{\mathrm{cap}}=\frac{\mu \varepsilon }{K}h\frac{dh}{dt}+\rho \mathrm{gh}$$

In Equation (4), the first term on the right-hand side refers to viscous resistance based on Darcy’s law while the second term represents the effect of gravity. *g* is the gravitational acceleration. Equation (4) can be modified as follows:

(5) $$\frac{dh}{dt}=\frac{\Delta P_{\mathrm{cap}}K}{\mu \varepsilon }\cdot \frac{1}{h}-\frac{\rho \mathrm{gK}}{\mu \varepsilon }$$

Equation (5) shows that there is a linear correlation between the capillary rising rate (d*h*/d*t*) and the reciprocal height of liquid (*h*^−1^). Thus, when the measured correlation between d*h*/d*t* and *h*^−1^ is regressed linearly, *K* and Δ*P*_cap_·*K* can be quantified from the intercept and slope of the regression line. Δ*P*_cap_ can be evaluated by dividing Δ*P*_cap_·*K* by *K*.

## 3. Results

### 3.1. Mono-Porous Al with Various Porosity

**Figure 3:**
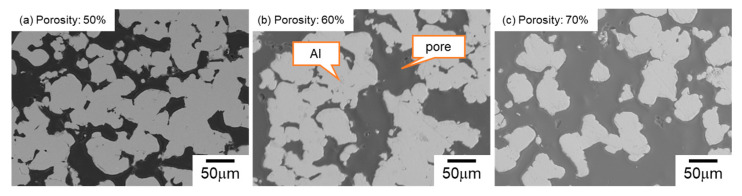
Representative SEM images of mono-porous Al: (**a**) S50, (**b**) S60, and (**c**) S70.

Figure 3 displays the SEM images of the microstructures of mono-porous Al with various volume fractions of small NaCl. The dark areas indicate the resin infiltrating into the pores and the bright areas represent the Al part. The shape of the pores was different from the original shape of the small NaCl particles (30–50 μm). The Al cell wall exhibited a particulate morphology, indicating that sintering was inhibited. Comparing with our previous work [27], where the large NaCl (330–430 μm) were used, the shape of the pores replicated the shape of the large NaCl particles, and the dense Al cell wall was formed. It was suggested that the particle size ratio of NaCl and Al (<45 μm) powders affected the pore morphology. When the NaCl particles were much larger than Al particles, the Al particles surrounded the NaCl particles and easily contacted adjacent Al particles, resulting in the forming of a dense cell wall via spark plasma sintering. In contrast, when the NaCl particle size was comparable to Al particles, the contacts between the Al particles were inhibited by larger fractions (50–70%) of NaCl particles. As a result, the Al cell wall was particulate, and the pores did not replicate the shape of NaCl particles. Even in sample S50, fabricated using small NaCl particles at a 50% volume fraction, it was confirmed through mass measurement that space holders were completely removed. As the volume fraction of NaCl increased from 50% to 70%, more small pores interconnected with each other. Consequently, the average pore size measured via image analysis increased from 35.2 to 148.3 μm. These results indicated that when the NaCl particle size was similar to the Al powder size, changing the volume fraction of NaCl could not only control the porosity but also vary the pore size. 

**Figure 4:**
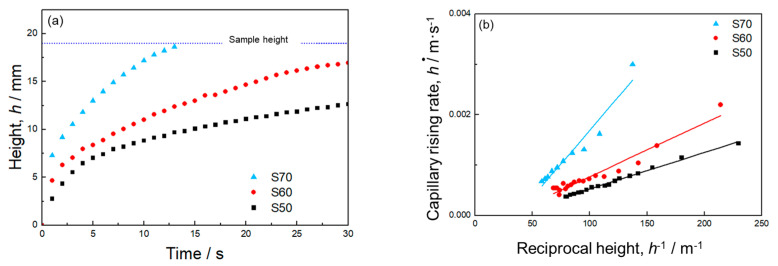
(**a**) Time evolution of the capillary rising height of mono-porous Al. (**b**) Relationship between capillary rising rate and reciprocal height.

Figure 4a presents the time evolution of the capillary rise height captured by the IR camera. In all the samples, the infiltrated water height sharply increased at the early stage of infiltration because of a small gravity effect. The rising rate slowed down as the influence of gravity became significant. The sample with larger porosity had a faster capillary rising rate. Infiltrated water reached the sample height within 15 s in sample S70. In samples S60 and S50, the water height continuously increased for 30 s and did not reach the sample height or equilibrium infiltration height at which the capillary force was balanced with the gravity during the measurement period. 

Figure 4b shows the relationship between the capillary rise rate (d*h*/d*t*) and the reciprocal height (*h*^−1^). In this figure, the data point at the final stage of the infiltration into sample S70 were excluded to eliminate the effect of water reaching the sample height on the capillary rise rate. There are good linear correlations between d*h*/d*t* and *h*^−1^, indicating that the capillary pressure and permeability can be quantified using Equation (5). 

The calculated values of Δ*P*_cap_, *K*, and Δ*P*_cap_·*K* are provided in Table 2. *K* was much more sensitive to porosity than Δ*P*_cap_: with increasing the porosity from 50% to 70%, *K* increased by approximately 7 times (1.1 × 10^−11^ m^2^ to 7 × 10^−11^ m^2^) while Δ*P*_cap_ decreased by 12.5% (360 to 315 Pa). Consequently, the capillary performance (Δ*P*_cap_·*K*) was enhanced by approximately 5 times when the porosity increased from 50% to 70%. As mentioned above, increasing porosity from 50% to 70% increased the pore size from 35.2 μm to 148.2 μm (Figure 3). According to Equation (1), the larger pore size reduced the capillary pressure. However, based on Equation (2), the permeability was improved by increasing the porosity and pore size. Thus, mono-porous Al fabricated using small NaCl particles exhibited the trade-off relation between the capillary pressure and permeability via the porosity. This trend was consistent with the results of mono-porous Al fabricated using large NaCl particles [27].

### 3.2. Bi-Porous Al with Various Volume Fractions of Large and Small Pores under a Constant Total Porosity

**Figure 5:**
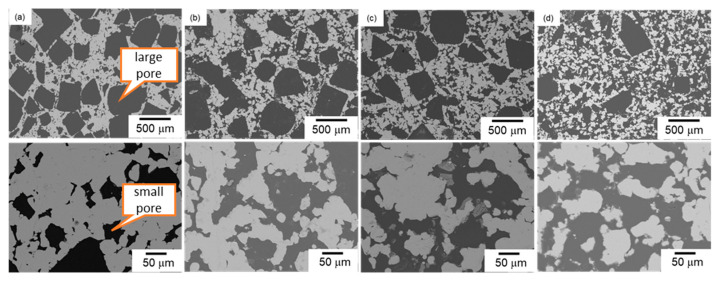
SEM images of bi-porous Al: (**a**) L60S10, (**b**) L50S20, (**c**) L40S30, and (**d**) L30S40.

Figure 5 shows the SEM images of the microstructures of bi-porous Al denoted as samples (a) L6010, (b) L50S20, (c) L40S30, and (d) L30S40. A typical bi-porous structure was successfully fabricated using NaCl space holder particles of two different sizes. The total porosity measured (listed in Table 1) was around 70%, which confirmed the removal of all the NaCl particles. In low-magnification images, cuboidal pores replicating the shape of large NaCl particles (Figure 1) were observed. The cuboidal large pores were less observed and became isolated when the volume fraction of large NaCl particles was decreased. In high-magnification images, although the shape of the small pores was not cuboidal, the size of the small pores was comparable to that of the small NaCl particles. When the volume fraction of small NaCl particles was increased, the small pores interconnected with each other. The area fraction of small pores in the cell wall of large pores was measured using image analysis and quantified at 23.4% for L60S10, 42.7% for L50S20, 47.6% for L40S30, and 52.8% for L30S40. The designed volume fraction of small pores in the cell wall was 25%, 40%, 50%, and 57%, respectively. The measured area fractions of the small pores were almost consistent with the designed volume fraction. These results demonstrated a high controllability of bi-porous structure using NaCl space holder particles with different particle sizes.

Figure S1 shows SEM images of feature pore structures of bi-porous Al. The small pores in the cell wall acting as the interconnected channels between the big pores can be observed, which guarantees an open-cellular structure between the big pores and helps improve the capillary performance of the bi-porous structure significantly.

**Figure 6:**
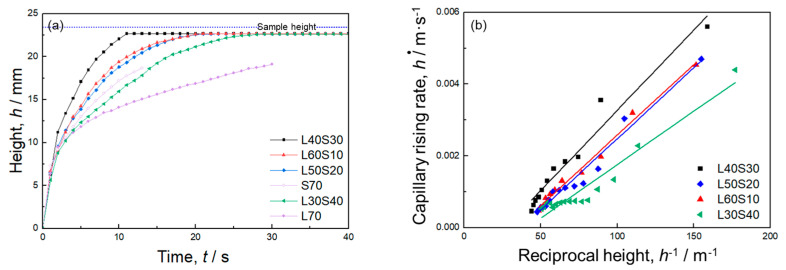
(**a**) Time evolution of the capillary rising height of bi-porous Al. (**b**) Relationship between capillary rising rate and reciprocal height.

Figure 6a shows the time evolution of the capillary height of four bi-porous samples measured by the IR camera. For comparison, two mono-porous samples, L70 [27] and S70 (Section 3.1), with the same total porosity as the bi-porous samples, are also presented in this figure. All the samples exhibited typical infiltration height curves, on which the infiltration height increased sharply at first and then slowly with infiltration progress. Finally, the infiltration height tended to saturate as the water reached the top of the sample. For the four bi-porous samples with constant total porosity, the increase in the fraction of small pores did not monotonously enhance the rising rate. The sample L60S10 exhibited a much higher capillary rising rate than the sample L70, indicating that replacing 10% large pores with small pores enhanced the capillary performance. The capillary rising rate of sample L50S20 was almost comparable to that of sample L60S10. Sample L40S30 exhibited a further improved capillary rising rate compared to samples L50S20 and L60S10. However, the capillary rising rate of sample L30S40 was smaller than that of sample L40S30 and was almost comparable to sample S70. These results indicated that bi-porous structures did not always exhibit better capillary performance than mono-porous structures and needed to be optimized. 

Figure 6b shows the relationship between the capillary rising rate (d*h*/d*t*) and reciprocal height (*h*^−1^) of the bi-porous samples. In the cases of samples L40S30 and L60S10, there are almost linear correlations between d*h*/d*t* and *h*^−1^ (R^2^ = 0.96 for L40S30 and 0.99 for L60S10). The capillary pressure and permeability of these samples can be obtained based on Equation (5). Samples L50S20 and L30S40 exhibited non-linear correlations between d*h*/d*t* and *h*^−1^, suggesting that the capillary performance changes with the infiltration progress. However, the deviation from regressed lines was small (R^2^ = 0.97 for L50S20 and 0.93 for L30S40). Analyzing the regression lines based on Equation (5) can provide an average of the capillary pressure and permeability throughout the overall infiltration into these samples. The quantified values of Δ*P*_cap_, *K*, and Δ*P*_cap_·*K* are provided in Table 2. 

**Figure 7:**
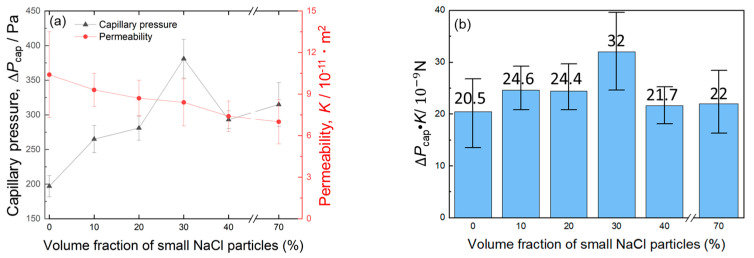
Change in (**a**) permeability, capillary pressure, and (**b**) their product with the volume fraction of small NaCl particles.

Figure 7a shows changes in the capillary pressure and permeability with the volume fraction of small NaCl particles (corresponding to the volume fraction of small pores). The capillary pressure first increased when the volume fraction of small pores increased from 0% to 30% and decreased when the volume fraction of small pores was 40%. Compared to sample L30S40, sample S70 exhibited a slightly higher capillary pressure. As a result, the sample L40S30 exhibited the highest capillary pressure of 381 Pa. The permeability kept decreasing slightly from 10.4 × 10^−11^ m^2^ to 7 × 10^−11^ m^2^ as the volume fraction of small pores increased. Thus, tailoring the bi-porous structure using two-sized space holder particles enhanced the capillary pressure while minimizing the decrease in permeability.

The capillary performance (Δ*P*_cap_·*K*) was also evaluated, as shown in Figure 7b. Samples L40S30 presented the highest Δ*P*_cap_·*K*, followed by L50S20, L60S10, S70, L30S40, and L70. This trend was consistent with the trend in capillary height curves in Figure 6a. Samples L40S30, L50S20, and L60S10 exhibited higher Δ*P*_cap_·*K* than mono-porous samples L70 and S70. It was confirmed that the bi-porous structure can improve capillary performance. In particular, the optimized sample L40S30 exhibited approximately 1.5 times higher capillary performance than the mono-porous samples.

Figure S2 displays the size distribution of small pores on cell walls of the large pores with different volume fractions of small NaCl. When the volume fraction was 10% (Figure S2a), most of the pores had smaller than 20 μm, but pores of sizes > 50 μm also existed. On the other hand, when the volume faction was 20% (Figure S2b) and 30% (Figure S2c), pore size had a concentrated distribution in 20–60 μm, and pores of sizes > 100 μm were formed. Thus, as the volume fraction of small NaCl increased, the small pore size increased and the distribution apparently widened and shifted to the large size. However, the effect of size distribution on the capillary performance cannot be discussed from these results because the volume fraction of large and small pores and the average pore size were changed at the same time. To discuss the effect of the pore size distribution on the capillary performance, it is necessary to fabricate the samples with various pore size distribution and a constant porosity and average pore size. This can be conducted in future works.

**Figure 8:**
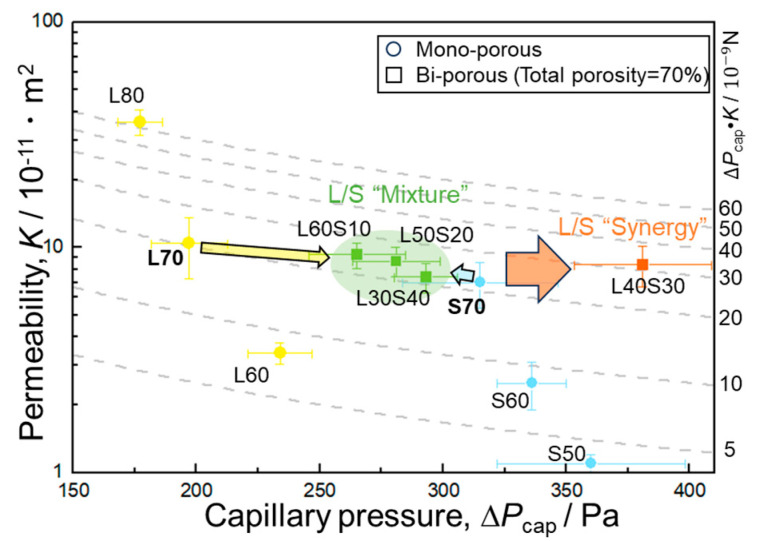
Plot of permeability and capillary pressure of mono-porous and bi-porous Al.

In Figure 8, the capillary pressure and permeability of all the samples evaluated in this study are plotted. For comparison, the properties of mono-porous Al fabricated using the large NaCl particles [27] are also shown in this figure. The dotted lines indicate isolines of the capillary performance (Δ*P*_cap_·*K*). As mentioned in Section 3.1, mono-porous samples exhibited the trade-off relationship between Δ*P*_cap_ and *K* via the porosity. When the porosity was the same, decreasing space holder size (pore size) increased the capillary pressure and decreased the permeability, resulting in almost constant capillary performance regardless of the pore size (compare L70 with S70 and L60 with S60). This trend was a well-known trade-off relation between Δ*P*_cap_ and *K* via the pore size. 

When the bi-porous samples were focused on, the capillary pressure and permeability of samples L60S10, L50S20, and L30S40 ranged from 265 to 293 Pa and 7.4 × 10^−11^ m^2^ to 9.3 × 10^−11^ m^2^, respectively, which were located between those of samples L70 (Δ*P*_cap_ = 197 Pa, *K =* 10.4 × 10^−11^ m^2^) and S70 (Δ*P*_cap_ = 315 Pa, *K =* 7 × 10^−11^ m^2^). Roughly speaking, these samples can be considered as mixtures of samples L70 and S70, although Δ*P*_cap_·*K* of the samples L60S10 and L50S20 was slightly higher than that of mono-porous samples. In contrast, sample L40S30 deviated far from the mixture-like properties. The capillary pressure of sample L40S30 (Δ*P*_cap_ = 381 Pa) was much higher than those of samples L70 and S70 while maintaining the permeability (*K =* 8.4 × 10^−11^ m^2^). It was considered that the large and small pores exhibited a synergy to enhance the capillary pressure. Thus, the bi-porous structure can overcome the trade-off relationship between Δ*P*_cap_ and *K* but must be tailored so that the properties do not fall into mere mixture law.

## 4. Discussion

This present study fabricated the bi-porous Al using two different-sized NaCl space holder particles and evaluated its capillary performance. The effect of the volume fraction of small/large NaCl particles (corresponding to the porosity of small/large pores) on capillary pressure and permeability was investigated under a constant total porosity of 70%. The properties of bi-porous Al were compared to those of mono-porous Al fabricated using the small or large NaCl particles alone. Mono-porous Al exhibited the trade-off relation between the capillary pressure and permeability via the porosity (Table 2 and Figure 8). This was because increasing the NaCl volume fraction improved the connectivity between adjacent pores and formed a large network of pores (Figure 3). The large network worked as large pores (Table 2), resulting in the enhancement of the permeability but decreasing the capillary pressure. The bi-porous structure could be controlled by tailoring the volume fractions of small/large NaCl particles (Figure 5, Table 1 and Table 2). Tailoring the porosity of small/large pores under the constant total porosity had a significant effect on the capillary pressure and a slight effect on the permeability (Figure 7a). Although samples L60S10, L50S20, and L40S30 exhibited higher capillary performances than samples L70 and S70 (Figure 7b), samples L60S10 and L50S20 roughly fell into the mixture of samples L70 and S70. Sample L40S30 exhibited the highest capillary performance (Figure 7b) in this study and the synergy effect of samples L70 and S70 on the capillary pressure (Figure 8). The reason for the better properties of sample L40S30 is discussed here. 

**Figure 9:**
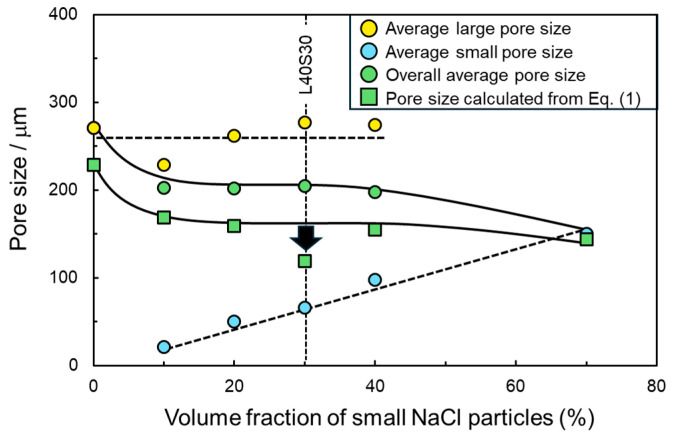
Changes in the average sizes of large, small, and overall pores as a function of the volume fraction of small NaCl particles. The flow channel size calculated from Equation (1) is also shown in this figure.

In Figure 9, average large, small, and overall pore sizes are plotted as a function of the volume fraction of small NaCl particles under a constant total volume fraction of NaCl of 70%. In addition, the effective flow channel size was inversely quantified using the measured capillary pressure (Table 2) and Equation (1). The surface tension of water (*s*_L_) at room temperature was 72 × 10^−3^ N/m, and the contact angle (*q*) between the water and Al was reported in the literature as 81° [30]. As the porosity of the small pores increased, the average large pore size remained nearly constant at around 260 μm while the average small pore size increased monotonously from 22.1 to 148.3 μm. As a result, the average overall pore size decreased by introducing small pores, became almost constant in the bi-porous samples, and decreased in sample S70. The effective flow channel size was also located between the average large and small pore sizes and varied with the same trend as the overall average pore size. This trend suggested that capillary pressure changed depending on the overall average pore size, which was consistent with L70/S70 mixture-like properties shown in Figure 8. However, the effective flow channel size of sample L40S30, which exhibited the highest capillary pressure (Figure 8), deviated from this trend. This suggested that the overall average pore size could not determine the capillary pressure of this sample, which corresponded to the noteworthy property compared to the other bi-porous sample shown in Figure 8.

Byon and Kim reported that the bi-porous wicks, which were fabricated by the sintering process using clusters of small-sized glass particles, exhibited better capillary performance than the mono-porous wicks [18]. They discussed the importance of the ratio of cluster size to particle size. When the ratio is in the range of 4–6, the capillary performance is improved because a liquid is wicked into small pores at first and improves the wettability between the liquid and solid (containing liquid in the small pores) at the large pores. When the ratio is lower or higher than this range, the capillary behaviors are close to the mono-porous wicks with large or small pores. The small pores in sample L40S30 might improve the wettability between water and solid cell walls of large pores and the capillary pressure. In Figure 9, the effective flow channel size was estimated from Equation (1), assuming that the contact angle between water and Al was constant at 81°. However, if wettability at the large pores was improved by water infiltration into small pores, the contact angle was not necessarily constant. The improved contact angle led to underestimating the effective flow channel size as shown in Equation (1).

**Figure 10:**
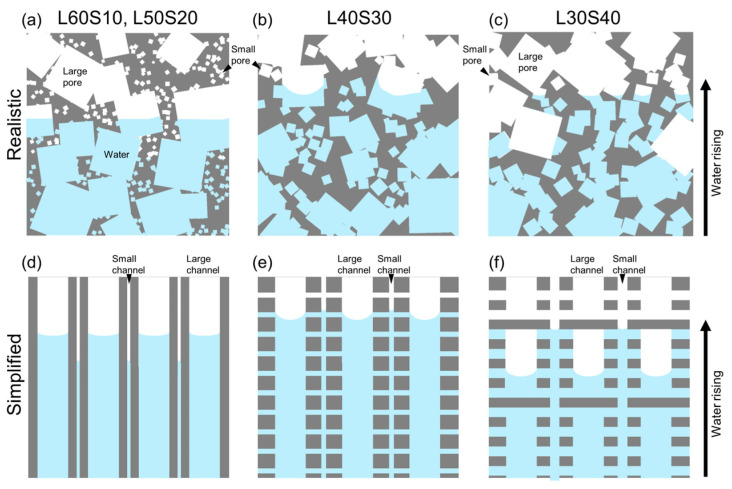
Hypothetical schematic illustration of capillary rising behaviors in (**a**) L60S10, L50S20, (**b**) L40S30, and (**c**) L30S40. The complex porous structures are simplified into straight channel models in (**d**–**f**).

The ratios of large pore size to small pore size were 10.3 for L60S10, 5.1 for L50S20, 4.2 for L40S30, and 2.8 for L30S40 (Table 2). Assuming that the ratio of cluster size to particle size can be replaced with the ratio of large pore size to small pore size, the size ratio of L50S20 and L40S30 was in the range of the appropriate ratio (4–6), supporting the better property of L40S30. Another important aspect of the bi-porous structure is the connectivity of two different-sized pores. In this study, the NaCl space holders were completely removed in all the samples, indicating the well-connected porous structure. However, the small pores need to be connected with both adjacent small and large pores to wet the cell wall of large pores and improve the wettability. The large pores also need to be connected to each other to ensure the main infiltration path has high permeability. These situations were not always established in all the samples. Specifically, the volume fraction of small pores in the cell wall of large pores in sample L60S10 was approximately 23.4%. The connection of the small pores with adjacent small and large pores was difficult to establish under such a small porosity. The volume fraction of large pores was approximately 30% in sample L30S40, in which the connection between adjacent large pores is difficult to establish. In addition, the capillary rising rate in small pores needs to be higher than that in large pores to improve the wettability because water infiltrating into the smaller pores must precede and wet the solid cell walls of the larger pores. The capillary rising rate at the early stage of infiltration is dominated by Δ*P*_cap_·*K* in Equation (1). As shown in Figure 8, the Δ*P*_cap_·*K* of the mono-porous samples with the same porosity (L70/S70 and L60/S60) was almost the same. Assuming that this situation was established in the bi-porous samples, the volume fraction of small pores in the cell wall needs to be higher than that of large pores. The volume fractions of small pores in the cell wall were 23.4% for L60S10, 42.7% for L50S20, 47.6% for L40S30, and 52.8% for L30S40. In samples L40S30 and L30S40, the volume fractions of small pores in the cell wall were higher than that of large pores, suggesting the preceding water infiltration into small pores. Thus, the superior properties of only the sample L40S30 were attributed to the appropriate pore size ratio, connectivity of small pores with both large and small pores, and the volume fraction of small pores in the cell wall higher than that of large pores. Figure 10 shows hypothetical schematic illustrations of capillary rising behaviors of bi-porous samples (a, d) L60S10, L50S20, (b, e) L40S30, and (c, f) L30S40. In samples L60S10 and L50S20, the large pores were well-connected to each other, whereas most of the small pores were connected with either adjacent large or small pores. Then, preceding water infiltration into the large pores was dominant for the capillary rising, and the samples did not benefit from the wetting of the cell walls of the larger pores via water infiltrating into smaller pores (Figure 10a). Based on this hypothesis, the situation is simplified, such as in Figure 10d, in which water infiltrates into large and small pores independently. In sample L30S40, the small pores became well-connected to each other, whereas the large pore network was separated. Then, water preferentially infiltrated into the small pores (Figure 10c). Although water infiltrating into the small pores improved the wettability at the large pores, poor connectivity between adjacent large pores resulted in infiltration into the large pores being significantly delayed due to infiltration into the small pores. Following this assumption, the situation is simply illustrated in Figure 10f. In sample L40S30, not only were the large pores well-connected to each other, but the small pores were also connected with adjacent large and small pores, allowing infiltration into both pores (Figure 10b). The slightly higher volume fraction of small pores in the cell wall (47.6%) compared to the volume fraction of large pores (40%) produced moderately preferential water infiltration in the small pores. Water infiltrating into the smaller pores then promoted improved wettability and meniscus formation within the larger pores, improving the capillary pressures within the larger pores. This situation is simplified in Figure 10e. However, these mechanisms were not completely demonstrated. To elucidate the mechanism of the superior properties in more depth, it is required to evaluate the capillary performance of the samples in which the bi-porous structures are further controlled (e.g., various fractions of large and small pores under a constant small pore size) and to observe the capillary rising behaviors directly.

## 5. Conclusions

In this study, bi-porous structures were controlled by sintering aluminum (Al) powder with sodium chloride (NaCl) space holder particles of two different sizes. The capillary pressure (Δ*P*_cap_), permeability (*K*), and their product (Δ*P*_cap_·*K*) were investigated and compared to that of mono-porous Al. The major conclusions are as follows:

(1) Mono-porous Al produced using small NaCl particles (30–50 μm) exhibited the trade-off relation between Δ*P*_cap_ and *K* via the porosity. This trend was consistent with the previous results of mono-porous Al fabricated using large NaCl particles (330–430 μm). The pores were well-connected to form large pores as the porosity increased, resulting in the trade-off relation.
(2) The mono-porous Al with a smaller pore size exhibited a higher Δ*P*_cap_ and a lower *K* compared to the mono-porous Al with a larger pore size under a constant porosity. This trend demonstrated the well-known trade-off relation between Δ*P*_cap_ and *K* via the pore size.
(3) The volume fractions of large and small pores in the bi-porous Al were successfully controlled under a constant total porosity of 70% by tailoring the blending volume fractions of large and small NaCl particles. Increasing the volume fraction of small pores from 0% to 30% increased the Δ*P*_cap_ from 197 to 381 Pa while slightly decreasing *K* from 10.4 × 10^−11^ m^2^ to 8.4 × 10^−11^ m^2^. When the volume fraction of small pores was increased to 40%, Δ*P*_cap_ and *K* degraded to 293 Pa and 7.4 × 10^−11^ m^2^.
(4) Almost all the bi-porous samples exhibited intermediate Δ*P*_cap_ and *K* between the mono-porous samples with large and small pores alone. However, the optimized bi-porous structure with 40% large and 30% small pores exhibited a higher Δ*P*_cap_ and Δ*P*_cap_·*K* than the mono-porous samples. The bi-porous structure was not always superior to the mono-porous structure and must be controlled to improve Δ*P*_cap_ and Δ*P*_cap_·*K*.

## Figures and Tables

**Table 1:** The blending volume fraction of large and small NaCl particles for fabricating mono-porous and bi-porous samples. The total porosity quantified by measuring the mass, diameter, and height of each sample is also shown.

	Sample	330~430 μm NaCl Volume Fraction (%)	30~50 μm NaCl Volume Fraction (%)	Designed Total Porosity (%)	Measured Total Porosity (%)
Mono-porous	S50	0	50	50	48.9
	S60	0	60	60	59.9
	S70	0	70	70	68.7
Bi-porous	L60S10	60	10	70	70.5
	L50S20	50	20	70	72.3
	L40S30	40	30	70	72.6
	L30S40	30	40	70	72.0

**Table 2:** Capillary performance of porous Al obtained in this study.

Sample	Average Large Pore Size (μm)	Average Small Pore Size (μm)	Permeability, $K/10^{-11}\;m^{2}$	Capillary Pressure, Δ*P*_cap_/Pa	Capillary Factor, $\Delta P_{\mathrm{cap}}\cdot K/10^{-9}\;$N
S50	-	35.2	1.1 ± 0.1	360 ± 38.0	4.0
S60	-	53.5	2.5 ± 0.6	336 ± 13.9	8.4
S70	-	148.3	7 ± 1.6	315 ± 31.6	22
L70	270	-	10.4 ± 3.1	197 ± 15.4	20.5
L60S10	228	22.1	9.3 ± 1.2	265 ± 19.6	24.6
L50S20	260	51.1	8.7 ± 1.3	281 ± 17.8	24.4
L40S30	276	65.7	8.4 ± 1.7	381 ± 28.0	32
L30S40	274	96.6	7.4 ± 1.1	293 ± 12.7	21.7

## Data Availability

Data will be made available on request.

## Supplementary Materials

The following supporting information can be downloaded at: https://www.mdpi.com/article/10.3390/ma17194729/s1, Figure S1: SEM images of feature pore structures of bi-porous Al: (a) L60S10, (b) L50S20, (c) L40S30, and (d) L30S40, Figure S2: Size distribution of small pores on cell walls of large pores: (a) L60S10, (b) L50S20, (c) L40S30, and (d) L30S40.
