# Supplementary material for: Enhancing Capillary Pressure of Porous Aluminum Wicks by Controlling Bi-Porous Structure Using Different-Sized NaCl Space Holders
Source: Materials (Basel). 2024 Sep 26;17(19):4729. doi: 10.3390/ma17194729 (PMC11477853; doi:10.3390/ma17194729)
Supplement: Supplementary file 1 [file materials-17-04729-s001.zip › materials-3226346-supplementary.pdf]

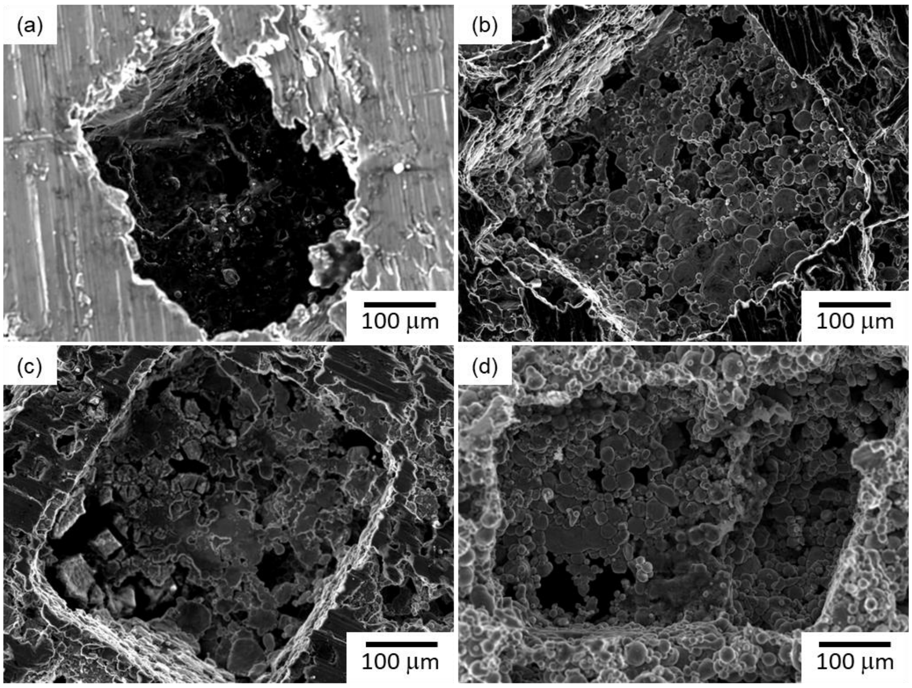


**Figure S1**. SEM images of feature pore structures of bi-porous Al: (a) L60S10, (b) L50S20, (c) L40S30, and (d) L30S40.

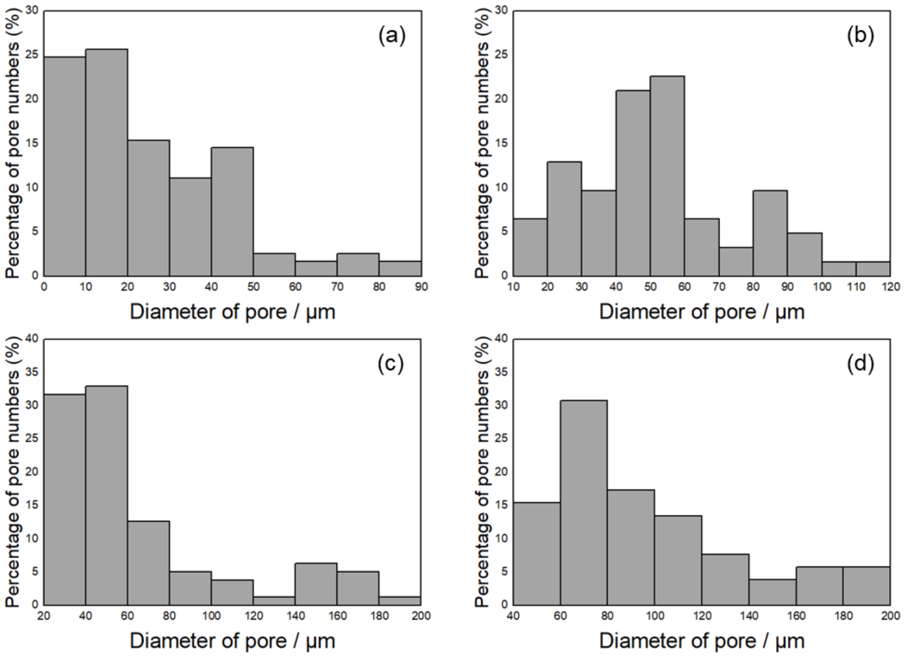


**Figure S2**. Size distribution of small pores on cell walls of large pores: (a) L60S10, (b) L50S20, (c) L40S30, and (d) L30S40.
